# Unilateral conductive hearing loss due to hydrocystoma in the external auditory canal: A rare case report

**DOI:** 10.1016/j.bjorl.2025.101564

**Published:** 2025-02-05

**Authors:** Francisco Campelo da Fonseca Neto, Renan Vieira de Brito, Eder Barbosa Muranaka, Arthur Menino Castilho

**Affiliations:** Universidade Estadual de Campinas (UNICAMP), Departamento de Otorrinolaringologia e Cirurgia de Cabeça e Pescoço, Campinas, SP, Brazil

**Keywords:** Hydrocystoma, External auditory canal, Unilateral conductive hearing loss, Ear canal cyst

## Introduction

The External Auditory Canal (EAC) is composed of cartilaginous tissue in its lateral third, covered by a thin epithelium adhered to the perichondrium with slight subcutaneous cellular tissue. The skin of the EAC contains secretory annex glands: eccrine (common sweat glands), apocrine (specialized sweat glands), and holocrine (sebaceous glands). The latter two are responsible for the production of cerumen, which promotes an acidic pH and acts against the proliferation of infectious agents, while the former is rarely found in the EAC.^1^

Hidrocystomas are benign cystic lesions characterized by the proliferation of sweat glands exhibiting eccrine or apocrine differentiation. While commonly found on the periorbital face as translucent or skin-colored papules and nodules,^2^ their occurrence within the EAC is exceedingly rare. Recognizing such atypical presentations is crucial for otorhinolaryngologists due to the potential for misdiagnosis and subsequent management challenges. This report presents a rare case of EAC hidrocystoma, emphasizing its clinical, radiological, and histopathological features, and discusses the diagnostic and therapeutic implications.

## Case report

A 43-year-old female patient, with no significant past medical history, presented with a one-year history of unilateral right-sided hearing loss. Otoscopic examination of the right ear revealed a narrowed EAC with a rounded bulge in its posterior portion, covered by intact skin without signs of inflammation, and a translucent tympanic membrane. No hyperemia, bulging, or visible lesions were observed on the tympanic membrane or the right auricle. Palpation of the right auricle did not elicit pain or discomfort. Otoscopy of the left ear was unremarkable.

**Fig. 1:**
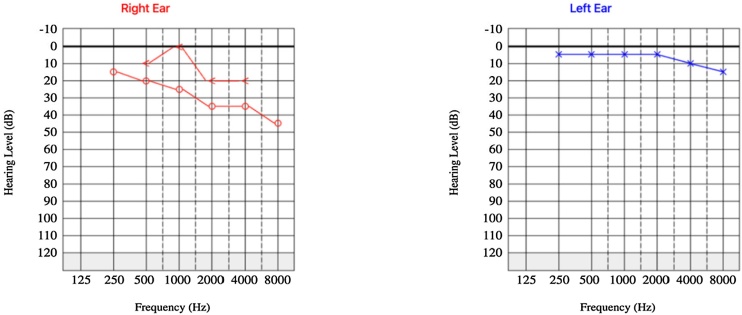
Preoperative audiometry demonstrating moderate unilateral conductive hearing loss.

**Fig. 2:**
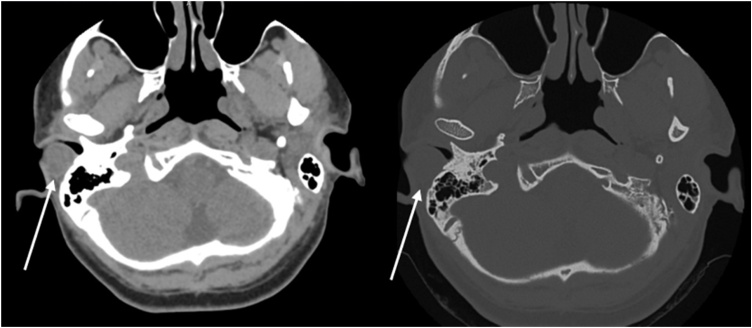
CT scan shows a lesion (arrow) along the posterior wall of the right external auditory canal, in contact with the mastoid.

Audiometric evaluation demonstrated mild right-sided conductive hearing loss and normal hearing in the left ear (Fig. 1). Computed Tomography (CT) of the temporal bones revealed a well-circumscribed formation adjacent to the posterior wall of the right EAC, measuring approximately 18 × 14 × 13 mm (Fig. 2). The lesion exhibited an average density of +35 Hounsfield units, resided between the surrounding soft tissues, and contacted the lateral margin of the mastoid process. Notably, the lesion caused partial narrowing of the EAC lumen. No bony erosion was observed.

**Fig. 3:**
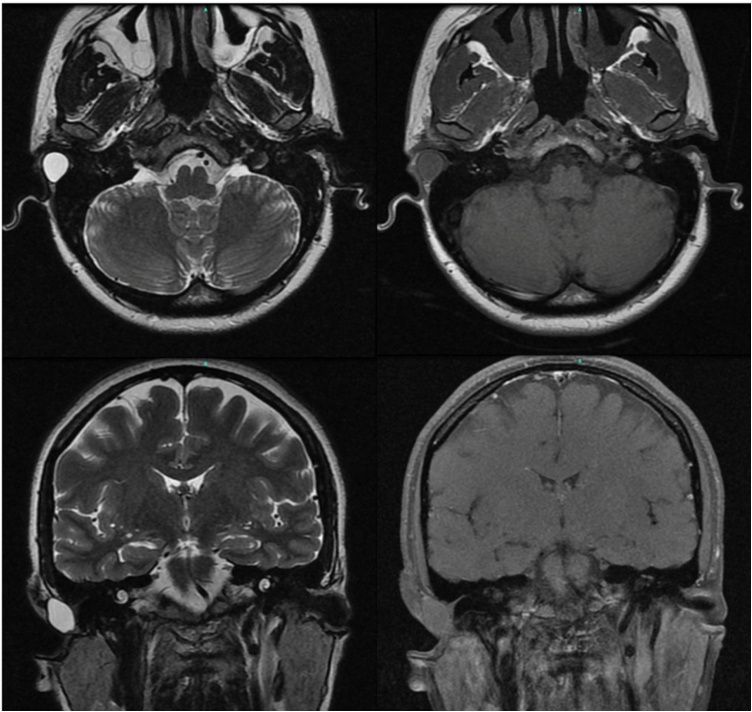
MRI shows a cystic lesion in the posterior-superior right external auditory canal, isointense on T1, hyperintense on T2, with no gadolinium enhancement or diffusion restriction.

Magnetic Resonance Imaging (MRI) of the posterior fossa was performed for further evaluation. The MRI demonstrated a well-defined cystic lesion in the posterosuperior aspect of the right EAC, measuring approximately 14.5 × 12.5 × 14 mm (Fig. 3). The lesion appeared isointense to the cerebral parenchyma on T1-weighted images and hyperintense to the cerebral parenchyma on T2-weighted images. No pathological gadolinium enhancement or diffusion restriction was observed.

**Fig. 4:**
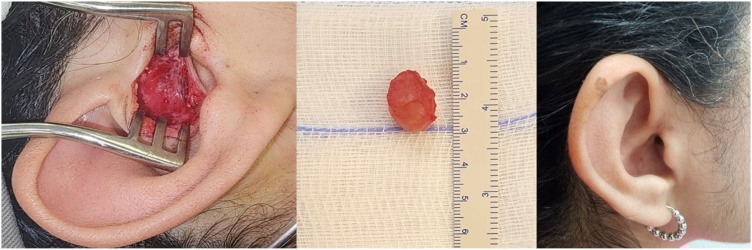
On the left, an intraoperative view of the lesion following incision in the tragus region. In the center, a soft, brownish nodular fragment encased by a whitish membrane. On the right, the postoperative appearance one week after resection.

Surgical resection of the lesion was performed without rupture of the cyst wall, and the excised material was submitted for histopathological analysis (Fig. 4). Gross examination revealed a soft, brownish nodular fragment measuring 18 × 13 × 12 mm, encapsulated by a whitish membrane. Sectioning revealed a smooth, brownish internal surface containing yellowish gelatinous material.

**Fig. 5:**
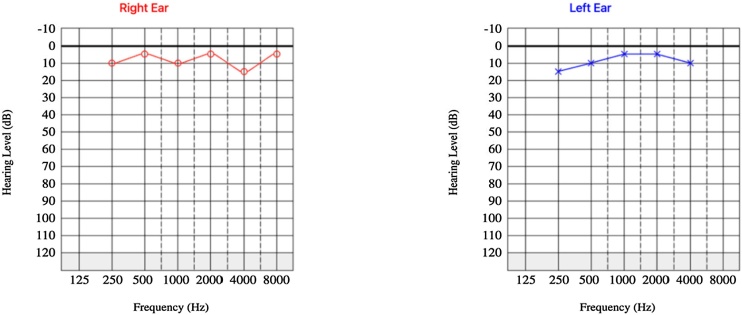
Postoperative audiometry demonstrating auditory recovery.

Microscopic examination demonstrated a cystic lesion lined by a double layer of epithelial cells without atypia. The cells of inner layer exhibited eosinophilic cytoplasm and apocrine-type secretion, while outer layer consisted of basal cells. No signs of malignancy were identified, and the surgical margins were free of the lesion. Based on these findings, a diagnosis of hidrocystoma was rendered. Post-operative audiometry revealed complete auditory recovery (Fig. 5).

## Discussion

Hidrocystomas of the EAC are extremely rare occurrences described in the literature. Their etiology remains unknown, and they are considered benign adnexal skin tumors. These tumors can be classified based on their histogenic derivation into two types: apocrine and eccrine. Both types present as papular or nodular lesions with cystic content, occasionally bluish in color, and can be solitary or multiple, isolated or recurrent. They are more common in adult women aged 30–70 years, with the most frequent locations being the periorbital and malar regions. Due to the mass effect in the EAC, they can also be associated with conductive hearing loss, as described in previous reports. Eccrine hidrocystomas typically range from 1 to 6 mm in diameter, while apocrine hidrocystomas range from 3 to 15 mm, with the most common location being the upper eyelid near the epicanthus. When multiple, they are associated with focal dermal hypoplasia (Goltz-Gorlin syndrome).^3^

Their recognition is crucial for otorhinolaryngologists as they can present with a skin-colored mass in the EAC and conductive hearing loss, mimicking more common conditions such as ceruminous impaction, otitis externa, inclusion cysts, mucous cysts, melanoma, pigmented basal cell carcinoma, hemangioma, and cholesterol granuloma. This case highlights the importance of considering hidrocystoma in the differential diagnosis of EAC masses, particularly in patients presenting with conductive hearing loss and preserved acoustic reflexes.^4^

The conductive hearing loss observed in this case can be attributed to the partial obstruction of the External Auditory Canal (EAC) by the hidrocystoma, which mechanically impaired sound conduction and primarily affecting high frequencies.

While CT scans can identify the presence of a mass, MRI, as employed in this case, provides superior soft tissue characterization, allowing for better delineation of the cystic nature of the lesion and aiding in its differentiation from other entities. The MRI findings in this case, including T1 hypointensity, T2 hyperintensity, and lack of contrast enhancement, are consistent with previous reports.^5^

Although imaging plays a crucial role in the initial evaluation, histopathological examination remains the gold standard for definitive diagnosis.^5^ The characteristic double layer of epithelial cells lining the cyst, observed in this case and consistently described in the literature, confirms the diagnosis and excludes other possibilities such as basal cell carcinoma. However, histological differentiation between apocrine and eccrine types can be challenging, and the presence of decapitation secretion and/or eosinophilic cytoplasm suggests apocrine differentiation.^1^

Surgical excision is the mainstay of treatment for solitary hidrocystomas of the EAC. However, in patients with recurrent lesions or those with difficult surgical access, alternative treatments described in the literature include topical atropine, scopolamine cream, botulinum toxin injection, and CO_2_ laser ablation.^3^

## Conclusion

This case underscores the importance of including hidrocystoma in the differential diagnosis of patients presenting with EAC masses and conductive hearing loss. Further studies are needed to clarify pathogenesis and improve diagnosis of this rare condition.

## Declaration of generative AI and AI-assisted technologies in the writing process

During the preparation of this manuscript, the authors utilized Google Gemini to enhance the quality of writing and ensure that the language is more comprehensive and appropriately aligned with scientific understanding. After using this tool, the authors reviewed and edited the content as needed and takes full responsibility for the content of the publication.

## Funding

There is no financial or material supports.

## Declaration of competing interest

The authors declare no conflicts of interest.
